# The effect of smoke-free legislation on the mortality rate of acute myocardial infarction: a meta-analysis

**DOI:** 10.1186/s12889-019-7408-7

**Published:** 2019-09-18

**Authors:** Min Gao, Yanyu Li, Fugang Wang, Shengfa Zhang, Zhiyong Qu, Xia Wan, Xiaohua Wang, Jie Yang, Donghua Tian, Weijun Zhang

**Affiliations:** 1 School of Social Development and Public Policy (SSDPP), Beijing Normal University, 19, XinjiekouWai Street, Beijing, 100875 China; 2 School of Humanities and Social Sciences, North China Electric Power University, Baoding, 071000 China; 3 People’s Bank of China Jinan Branch, Jinan, 250021 China; 4 Institute of Basic Medical Sciences, Chinese Academy of Medical Sciences and School of Basic Medicine, Peking Union Medical College, Beijing, 100005 China; 5 Tobacco control office, Chinese Center for Disease Control and Prevention (China CDC), Beijing, China

**Keywords:** Smoke-free legislation, Acute myocardial infarction (AMI), Systematic review, Meta-analysis

## Abstract

**Background:**

Several studies have demonstrated that smoke-free legislation is associated with a reduced risk of mortality from acute myocardial infarction (AMI). This study aimed to examine and quantify the potential effect of smoke-free legislation on AMI mortality rate in different countries.

**Methods:**

Studies were identified using a systematic search of the scientific literature from electronic databases, including PubMed, Web of Science, ScienceDirect, Embase, Google Scholar, and China National Knowledge Infrastructure (CNKI), from their inception through September 30, 2017. A random effects model was employed to estimate the overall effects of smoke-free legislation on the AMI mortality rate. Subgroup analysis was performed to explore the possible causes of heterogeneity in risk estimates based on sex and age. The results of meta-analysis after excluding the studies with a high risk of bias were reported in this study.

**Results:**

A total of 10 eligible studies with 16 estimates of effect size were included in this meta-analysis. Significant heterogeneity in the risk estimates was identified (overall I^2^ = 94.6%, *p* < 0.001). Therefore, a random effects model was utilized to estimate the overall effect of smoke-free legislation. There was an 8% decline in AMI mortality after introducing smoke-free legislation (RR = 0.92, 95% confidence interval (CI): 0.90–0.94). The results of subgroup analyses showed that smoke-free legislation was significantly associated with lower rates of mortality for the following 5 diagnostic subgroups: smoke-free in workplaces, restaurants and bars (RR = 0.92, 95% CI: 0.90–0.95), smaller sample size (RR = 0.92, 95% CI: 0.89–0.95), study location in Europe (RR = 0.90, 95% CI: 0.85–0.94), regional study area (RR = 0.92, 95% CI: 0.89–0.94), and no previous local smoke-free legislation (RR = 0.91, 95% CI: 0.90–0.93). However, there was not much difference in AMI mortality rates after the legislation between the longer (RR = 0.92, 95% CI: 0.86–0.98) and shorter follow-up duration subgroups (RR = 0.92, 95% CI: 0.89–0.94).

**Conclusion:**

Smoke-free legislation could significantly reduce the AMI mortality rate by 8%. The reduction in the AMI mortality rate was more significant in studies with more comprehensive laws, without prior smoke-free bans, with a smaller sample size, at the regional level, and with a location in Europe.

**Electronic supplementary material:**

The online version of this article (10.1186/s12889-019-7408-7) contains supplementary material, which is available to authorized users.

## Background

The widespread use of tobacco and secondhand smoke exposure had been problematic public health issues [1], which had greatly damaged human health. The *Global Burden of Disease Study (GBD) 2015* estimated that smoking was the second leading risk factor for attributable mortality among both men and women, and a total of 6.4 million deaths were attributable to smoking worldwide [2]. Secondhand smoke exposure was more likely to increase the negative health effects on passive smokers [3–5], even though low dose exposure could also increase cardiovascular risk by 25 to 30% [6]. *GBD 2013* estimated that secondhand smoke accounted for an additional 331,000 deaths and 9.3 million DALYs [7]. Several studies also showed that smoking may be an important independent risk factor for the development of myocardial infarction in male patients aged above 40 years old [8], in young adults [9], and in the Italian population [10]. Another study indicated that smoking significantly influenced the risk of first acute myocardial infarctions in a dose-dependent manner [11]. It is well known that China is the world’s largest consumer of tobacco producer and consumer, which accounts for about 40% of worldwide cigarette production and is home to a quarter of the world’s smokers. Therefore, China has a large smoking-related chronic disease burden, which is increasing further as China’s population ages. Furthermore, a recent study also showed that the implementation of tobacco control policies in China since the signing of the WHO Framework Convention on Tobacco Control in 2003 has not been effective in reducing smoking prevalence because of the factors about cultural context, economic and social barriers [12].

The effect of smoke-free legislation on the AMI mortality rate remains controversial. Some studies have shown that smoke-free legislation was significantly associated with a decline in AMI deaths [13, 14]. However, other studies did not find a significant decline in mortality due to AMI after legislation in North America, although the methodology concerning coverage of the local smoke-free legislation was questioned [15]. Another recent study by the U.S. National Center for Health Statistics indicated that the declines in AMI mortality in California (2.0%), Utah (7.7%) and Delaware (8.1%) were not significantly different from the expected declines. Furthermore, the AMI mortality rate increased by 8.9% in South Dakota after the ban [16].

Smoke-free legislation with different degrees of comprehensiveness has been implemented in several countries. However, the decreases in the AMI mortality rate following legislation varied across different countries. AMI deaths registered by the National Statistics Institute decreased by 9% for men and 8.7% for women after prohibiting smoking in all indoor workplaces in Spain, especially among people over 64 years of age [17]. In Massachusetts, USA, the AMI mortality rate decreased by 7.4% (95% CI:3.3–11.4) after implementation of the state law [14].

The decline in the AMI mortality rate following the implementation of smoke-free legislation may vary in different follow-up periods. The 2009 report published by the International Agency for Research on Cancer (IARC) demonstrated that the mortality risk of acute myocardial infarction showed the largest decline (10–20%) in the first year after the implementation of smoke-free legislation [18]. However, another meta-analysis showed that there was no association between the AMI risk reduction and smokefree laws increased with time [19].

A growing body of literature has explored the relationship between smoke-free legislation and the AMI mortality rate, and several new studies have assessed more comprehensive or less comprehensive smoke-free legislation (workplaces only; workplaces and restaurants; or workplaces, restaurants, and bars), extended follow-up durations, different study locations and whether a previous ban was in effect. However, the results so far have revealed a large variation in the effect sizes, ranging from − 9 to 23%. To more accurately estimate the effect of smoke-free legislation on the AMI mortality rate in the general population, this study identified relevant studies using a systematic search and meta-analysis. In addition, subgroup analysis was conducted to investigate difference in the effect sizes. Furthermore, we expected that the results of this study could provide more comprehensive evidence, based on previous studies, for promoting tobacco control legislation in China. This study followed the PRISMA guidelines and was registered in PROSPERO (CRD42016051951).

## Methods

### Search strategies

An electronic literature search was conducted using electronic databases including PubMed, Web of Science, ScienceDirect, Embase, Google Scholar, and CNKI (China National Knowledge Infrastructure), from their inception to September 30, 2017. Publications in both Chinese and English were included. The terms “smoke law (smoke legislation or smoke ban or smoke-free)”, “acute myocardial infarction (cardiovascular or coronary)”, and “mortality (death)” in the titles, abstracts, and keywords were used in this study. Additionally, the references cited in the selected articles were also searched manually. Study selection, risk of bias, and data extraction were accomplished by 2 reviewers, and the discrepancies were further resolved by consensus among the authors of this manuscript. More details of the search syntax are shown in Additional file 1.

### Study selection

Studies that met the following criteria were eligible: (1) measured the AMI mortality rate or death number at baseline, (2) reported the relative risk of the AMI mortality rate of the whole target population, and (3) published in English or Chinese. Furthermore, this review excluded the following types of studies: (1) those that only focused on AMI (coronary heart disease/ cardiovascular disease) incidence or hospital admissions, without AMI mortality rate information; (2) studies that only paid attention to practical measures to prohibit smoking, such as increasing the tobacco tax, advertising on the harmful effects of smoking, etc.; (3) studies for which the full text of the article could not be retrieved for full evaluation; (4) those that did not provide sufficient data about population size, the number of AMI deaths, etc.; (5) studies that were meta-analyses or review articles; (6) those that were not original or were cross-sectional studies; and (7) studies that did not provide estimates for the effect of smoke-free legislation on mortality rate for the whole target population.

**Fig. 1:**
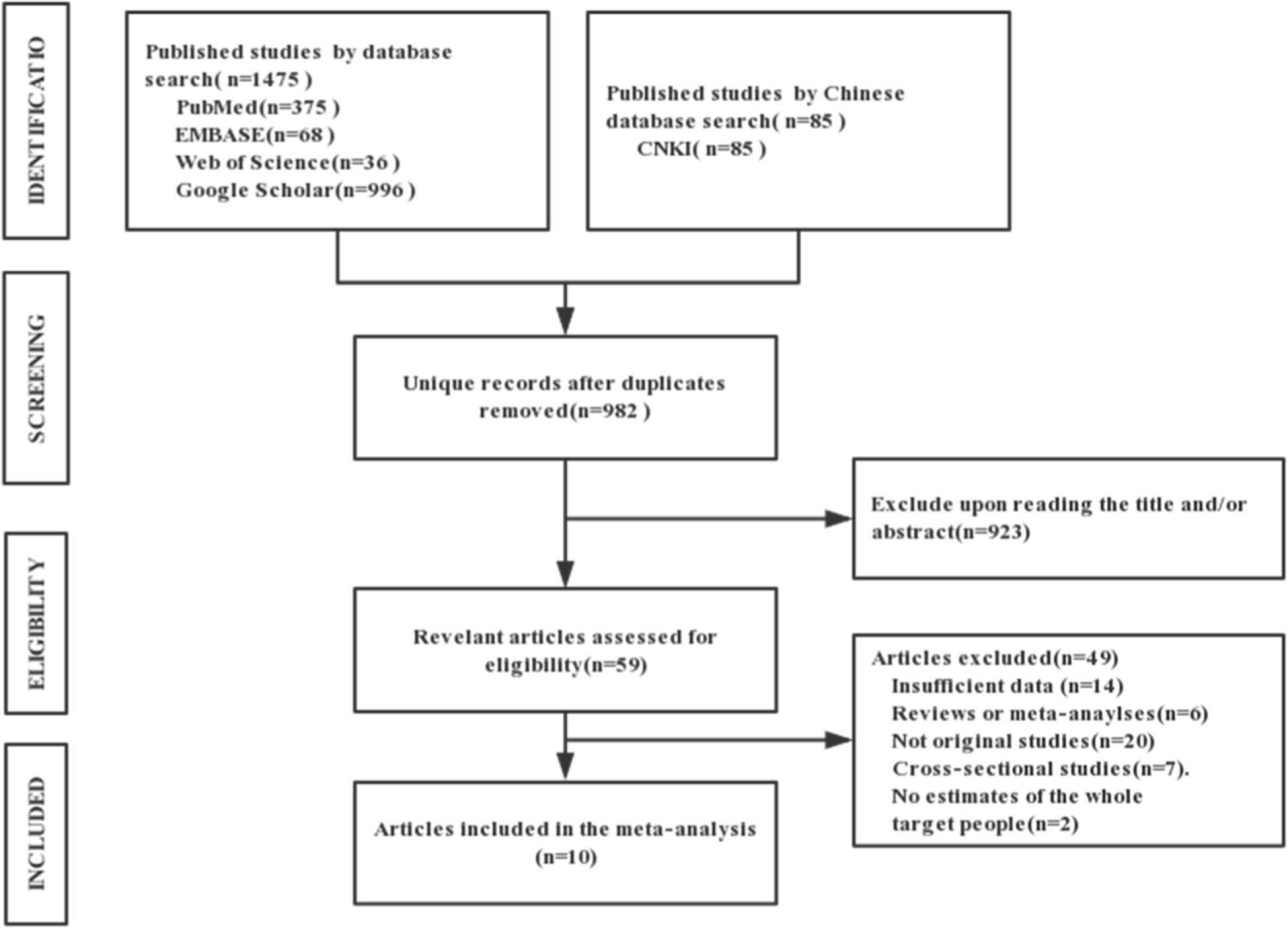
PRISMA chart for study identification

A total of 1560 articles were searched in all fields, including 375 articles from PubMed, 36 articles from Web of Science, 68 articles from Embase, 996 articles from Google Scholar, and 85 articles from CNKI. After removing duplicate articles, 982 articles were retrieved. Based on the exclusion criteria, 923 articles were excluded after reading the title and/or abstract. The remaining 59 articles were retrieved for full evaluation. Fourteen articles did not provide sufficient data about population size, the number of AMI deaths, etc. Six articles were meta- analyses or review articles, 20 studies were not original studies and 7 were cross-sectional studies. In addition, 2 studies did not provide estimates for the effect of smoke-free legislation on the mortality rate of the whole target population (Fig. 1). Ultimately, 10 articles were identified in this study.

### Data extraction and risk of bias

For each study, the following information was retrieved: study location, research classification, effective date of smoke-free legislation, study period, target population, comprehensiveness of the law, previous ban in place, population at risk, number of AMI deaths, RR value, 95% confidence interval, AMI definition, sources of data, measures/statistical methods and control variables. Seven studies provided relative risk values of AMI mortality rate and corresponding 95% confidence interval [14, 15, 17, 20–23]. For studies that did not report an AMI mortality rate, the estimated effects (RRs) were calculated [16, 24, 25]. The detailed characteristics of the eligible studies are shown in Table 1.

**Table 1:** Information of Selected Studies

Author	Study location	National study	Effective date of a law	Study period	Target people	Comprehensiveness of a law	Previous ban in place	Size of Population at risk	AMI deaths cases	RR value	95% Confidence interval	AMI definition	Source of data	Measure /Statistical Method	Control variable
Europe															
Joan R Villalbí2011	Spain	Yes	January 1, 2006	January 12,004- December 31,2006	Ages ≥35 years	Workplaces (with exemptions for bars, cafes, restaurants, night clubs and discos)	Yes	–	68,862	0.90	0.88–0.92	ICD-10: CM 055	the National Statistics Institute (INE in its Spanish acronym)	Poisson regression	Age, gender
					Ages ≥35 years, male			–	40,080	0.90	0.88–0.93				
					Ages ≥35 years, female			–	28,782	0.90	0.87–0.92				
				January 1, 2004- December 31,2007	Ages ≥35 years			–	90,382	0.86	0.84–0.88				
					Ages ≥35 years, male			–	52,583	0.86	0.83–0.88				
					Ages ≥35 years, female			–	37,799	0.86	0.84–0.89				
FernandoAguero2013	Girona, Spain.	No	January 1, 2006	January 1, 2002-December 31, 2008	Ages 35–74 years	All indoor public places and workplaces but allowed some exceptions in hospitality venues	Yes	–	891	0.82	0.71–0.94	ICD-9: 410–414, 798; ICD-10:I20–I25, I513, R960, R961, R98, R99	The REGICOR Study, conducted in six counties of the Girona province in the north east of Spain	Poisson regression model	Age, sex, smoking status, existing trend, and seasonality
					Ages 35–74 years, female			–	200	0.72	0.52–0.97				
					Ages 35–74 years, male			–	691	0.85	0.72–0.99				
					Ages 35–64 years			–	359	0.94	0.76–1.17				
					Ages 65–74 years			–	532	0.74	0.62–0.89				
Sericea Stallings-Smith 2013	Ireland	Yes	March 29, 2004	January 1, 2000-December 31,2007	Ages ≥35 years	Workplaces including restaurants, bars, and pubs	Yes	1,900,000	–	0.97	0.92–1.02	ICD-9:410; ICD-10:I21	Central Statistics Office (CSO) Ireland.	Poisson linear regression model with interrupted time-series analysis	Time trend, season, influenza, and smoking prevalence
					Ages ≥35 years, male				–	0.97	0.91–1.02				
					Ages ≥35 years, female				–	0.97	0.91–1.03				
					Ages 35–64 years				–	0.97	0.86–1.07				
					Ages 65–84 years				–	0.94	0.89–1.00				
					Ages> 85 years				–	1.01	0.94–1.09				
United States															
Kanaka D. Shetty2011	United States	Yes	1995	1990–2004	All	All workplaces except bars and restaurants	Yes	–	2,018,548	1.02	0.99–1.05	ICD-9:410; ICD-10:I21	Multiple Cause of Death (MCD) database	Region-level fixed effects multivariate linear regression model	Secular trends and regions
Melanie S. Dove 2010	Massachusetts, US	No	July 2004	January 11,999- -December 31, 2006	Ages ≥35 years	All workplaces, including restaurants and bars	No	2,507,320	20,806	0.90	0.86–0.95	ICD-10:I21	Massachusetts Registry of Vital Records and Statistics	Poisson regression model adjusted for a linear time term	Long-term trend, season, particulate matter less than 2.5 lm aerodynamic diameter (PM_2.5_),10–12 and influenza
					Ages > 35 years		Yes	835,597	6176	1.01	0.92–1.11				
					Ages > 35 years		–	3,342,917	26,982	0.93	0.89–0.97				
					Ages > 35 years, male			1,548,463	13,595	0.95	0.89–1.01				
					Ages > 35 years, female			1,794,454	13,387	0.90	0.85–0.96				
					Ages 35–64 years			2,482,755	4162	0.92	0.82–1.04				
					Ages 65–75 years			427,830	4657	0.99	0.89–1.11				
					Ages> 75 years			432,332	18,163	0.91	0.86–0.96				
McAlister 2010^①^	Jefferson County, Texas, USA	No	Autumn of 2000	1996–2005	All	Unclear	–	250,000	–	0.84	0.77–0.91	ICD-10:I21	Texas Department of State Health Services	Bivariate piecewise linear regression model	None
Brad Rodu2012^②^	Utah, US	No	January 1, 1995	1991–1995	Ages > 45 years	Enclosed indoor places of public access, bars exempted	No	488,000	767	0.92	0.90–0.94	ICD-9:410; ICD-10:I21	Compressed Mortality File from the National Center for Health Statistics (NCHS)	Test for difference between two independent proportions	None
	California, US		January 1, 1995	1991–1995		Enclosed workplaces and restaurants without bars	Yes	9,100,000	17,656	0.98	0.97–0.99				
	South Dakota, US		July 1, 2002	1999–2003		Most workplaces, bars and casinos exempted	No	287,000	686	1.09	0.95–1.25				
	Delaware, US		November 27, 2002	1999–2003		Most indoor public places, including restaurants and bars	No	305,000	433	0.92	0.90–0.93				
	Florida, US		July 1, 2003	2000–2004		Most indoor public places, including restaurants and bars	No	720,000	10,073	0.91	0.91–0.92				
	New York, US		July 24, 2003	2000–2004		All workplaces, including restaurants and bars	Yes	720,000	10,347	0.88	0.83–0.93				
Carl Bartecchi 2006	Pueblo, United States	No	July 1, 2003	January 1, 2002 - December 31,2004	All	Inside the workplace and all buildings open to the public	Yes	147,751	–	0.77	0.64–0.93	ICD-9:410	Health Statistics Section of the Colorado Department of Public Health and Environment	Poisson regression model, with the test of linear contrasts between pre-ordinance and post-ordinance changes.	Time, location, time-by location interaction, and harmonics to account for seasonality
Other locations															
Tania 2016^③^	São Paulo city, Brazil	No	August 1, 2009	January 2005- December, 2010	All	Prohibited the use of cigarettes and other tobacco products in closed and semi-closed places, public and private, with the exception of residences, places of religious worship where smoking is part of the ceremony and sites designated for the consumption of tobacco products.	Yes	–	39,177	0.95	0.93–0.96	ICD-10: I21,I22,I23,I24	Mortality Information System (SIM)	Autoregressive Integrated Moving Average with exogenous variables (ARIMAX) with Interrupted Time Series Analysis (ITSA)	Total hospital admission, carbon monoxide, minimum temperature and air relative humidity
TQ Thach	Hong Kong, China	No	1 January 2007	1 January 2001–31 December 2011	All	Workplaces including restaurants, bars, and pubs	No	–	–	0.87	0.81–0.94	ICD-10:I21	Hong Kong Special Administrative Region (SAR) Government Census and Statistics Department	Poisson regression model	Age, gender

Note:①②③In these articles, the relative risk of AMI mortality were not provided. So based on provided AMI death, AMI mortality and population data, we calculated the RR value of AMI mortality

The data included in this study were derived from observational studies, which increases the risk of bias. A seven-domain Cochrane handbook evaluation was adopted to evaluate the risk of bias in the included studies [26]. The parameters for each study were graded: low risk of bias, moderate risk of bias, and high risk of bias, and an overall assessment for each study was determined. 3 articles showed a high risk of bias [15, 16, 24], 3 articles showed a moderate risk of bias [14, 20, 22] and 4 articles showed a low risk of bias [17, 21, 23, 25]. More details are presented in Table 2.

**Table 2:** Risk of bias assessment

Study, year	Intervention independent of other changes?	Shape intervention effect pre-specified?	Intervention unlikely to affect data collection?	Knowledge of allocated interventions adequately prevented?	Incomplete outcome data adequately assessed?	Free from selective outcome reporting?	Free from other risks of bias	Risk of bias
Villalbí 2011	High	Low	Low	Low	Low	Low	Low	Low
Fernando 2013	High	Low	Low	Low	Low	High	Low	Moderate
Sericea Stallings-Smith 2013	High	Low	Low	Low	Low	Low	Low	Low
Carl Bartecchi 2006	High	Low	Low	Low	Low	Low	High	Moderate
Shetty, K. D 2009	High	High	Low	Low	Low	Low	High	High
Dove 2010	High	Low	Low	Low	Low	High	Low	Moderate
McAlister 2010	High	Low	Low	Low	High	High	High	High
Brad Rodu 2012	High	Low	Low	Low	Low	High	High	High
Tania 2016	High	Low	Low	Low	Low	Low	Low	Low
TQ Thach 2016	High	Low	Low	Low	Low	Low	Low	Low

### Analysis strategy

All analyses were conducted using Stata. Q tests were employed to reveal heterogeneity among the selected studies (*P* = 0.000). Included studies were conducted in different countries; therefore, a random-effect meta-analysis was adopted to consider nonrandom variability of estimates among the included studies. Heterogeneity was quantified using the I^2^ statistic (inconsistency was defined as I^2^ > 50%). In this study, heterogeneity was significant in the random effects model (overall I^2^ = 94.6%, *p* < 0.001). Thus, funnel plots and Egger’s test were used to evaluate potential publication bias. In the absence of bias and between study heterogeneity, the scatter plot of the effect estimates from individual studies will resemble a symmetrical inverted funnel. However, funnel plot asymmetry should not be equated with publication bias, because publication bias does not completely explain the asymmetry, since many of the beneficial effects reported from smaller studies were not significant [27]. Eventually, Egger’s test was employed to statistically examine the symmetry. Furthermore, subgroup analyses were carried out to examine the robustness of this meta-analysis. The following characteristics, including study location (North America versus Europe versus others), study classification (national versus regional), post-ban follow-up duration (> 2 years versus ≤2 years), comprehensiveness of the law (workplaces, restaurants and bars versus workplaces only), number of AMI deaths (≥10,000 versus < 10,000), and previous ban in place (with prior local law versus without previous ban), could influence the results. Therefore, these characteristics were used to account for the heterogeneity (using the Stata metan procedure). Analyses were performed for each category, and overall relative ratios for each category were calculated, and then compared with the I^2^ statistic for heterogeneity. After excluding studies with a high risk of bias, an additional sensitivity analysis was also conducted to evaluate the impact of smoke-free legislation on the AMI mortality rate by gender and age. The following study characteristics, which could influence the results of this study, were also examined: effective date of smoke-free legislation, previous ban in place, risk of bias, post-ban follow-up duration, comprehensiveness of the law, number of AMI deaths, study location and study classification.

When the estimated effects (RRs) of AMI mortality were not provided in the studies, calculation of the RR value was required. The calculation procedures were as follows:

1 $$ {\mathrm{RR}}_1={\mathrm{N}}_{\mathrm{TA}}/{\mathrm{N}}_{\mathrm{TB}} $$

where the capital letter N represents the number of deaths, and the subscript letters TA and TB represent the duration before the ban and after the ban, respectively. The variance of the logarithm of RR_1_, V_1_ was estimated by

2 $$ {\mathrm{V}}_1=\left(1/{\mathrm{N}}_{\mathrm{TA}}\right)+\left(1/{\mathrm{N}}_{\mathrm{TB}}\right)\kern2.25em $$

with the lower and upper 95% CI of RR_1_ estimated by

3 $$ {\mathrm{RR}}_{l1},{\mathrm{RR}}_{u1}=\mathit{\exp}\left(\log {\mathrm{RR}}_1\pm Z\sqrt{{\mathrm{V}}_1}\right) $$

## Results

### Characteristics of the studies

A total of 1475 articles in English and 85 articles in Chinese were searched. The final set consisted of 10 eligible studies, including 2 articles conducted in Spain [17, 20], 1 article in Ireland [21], 5 articles in the US [14–16, 22, 24], 1 article in Brazil [25], and 1 articles in China [23]. The total number of participants was 2,266,256. All of these studies focused on the AMI mortality rate, and provided estimates for the effect of smoke-free legislation on the mortality rate. The details of the included studies are summarized in Fig 1.

Some studies provided multiple relative risks for different age and sex subgroups; thus, 16 estimates of relative risks from 10 eligible studies reported before September 30, 2017, were included in the meta-analysis. Table 1 shows the detailed characteristics of the 10 eligible studies. Of the 10 studies, 9 showed that the AMI mortality rate declined after introducing the legislation; the exception was1 study that did not provide evidence that smoke-free legislation could result in a measurable reduction in the AMI mortality rate [15]. There were 4 studies that followed up for 2 years or less after the enactment of smoke-free legislation [16, 17, 22, 25], and the other 6 studies provided information on AMI mortality and followed up for more than 2 years [14, 15, 20, 21, 23, 24]. Smoke-free legislations in workplaces only were described in 3 studies [15, 17, 20]. Six studies reported that smoke-free legislations were more comprehensive, including workplaces, restaurants and bars [14, 16, 21–23, 25]. Five studies had a large number of AMI death cases (≥10,000) [14–17, 25], and 3 studies were national studies [15, 17, 21], all of the data were obtained from national surveys. Five of them focused on North American smoke-free legislation [14–16, 22, 24], 1 of which reported results from 6 US regions [16]; 3 studies location were located in Europe [17, 20, 21];1 was in Brazil [25], and another 1 was in China [23] . Four studies indicated that there were relevant smoke-free policies before introducing the smoke-free legislation [15, 17, 22, 25]; 4 studies reported that there were no such policies [20, 21, 23, 24]. One study compared AMI mortality after the legislation with and without previous local smoke-free legislations [14], and 1 study did not provide exact information on whether previous local smoke-free legislations were in place [16]. All data were from official sources and were obtained from relevant academic authorities or hospitals.

### Meta-analysis

**Fig. 2:**
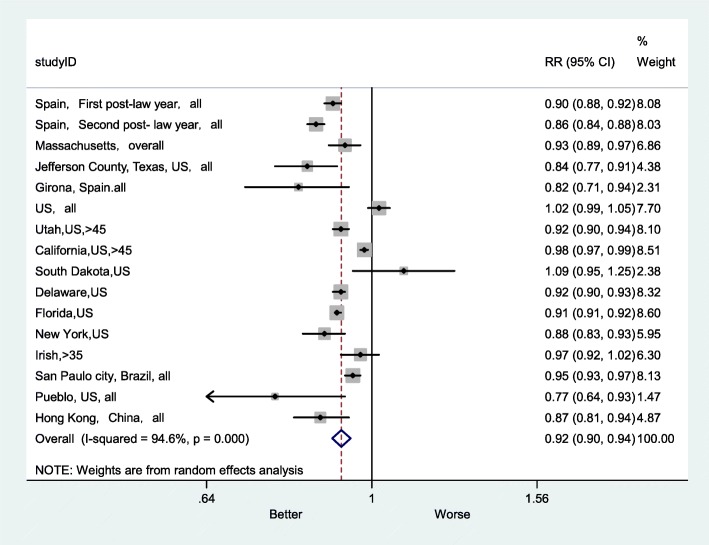
Forest plot of random effects meta-analysis of studies examining the effect of smoke-free legislation on AMI mortality

As shown in Fig. 2, comparison of the overall RR of AMI mortality before the smoke-free legislations with the RR after the smoke-free legislations, which was 0.92 (95% CI: 0.90–0.94), suggested that smoke-free legislation could reduce the AMI mortality rate by 8%.

**Fig. 3:**
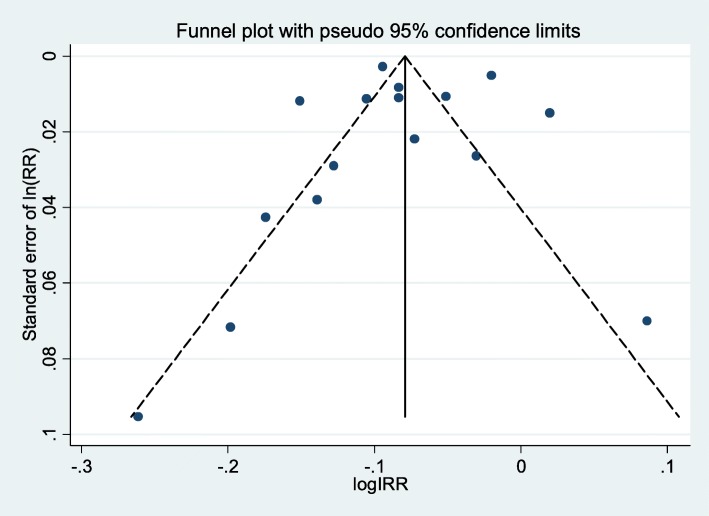
Funnel plot to illustrate possible publication bias

A funnel plot and Egger’s regression test were employed to examine publication bias (Fig. 3). The funnel plot appeared symmetrical, and Egger’s regression test reported that there was no publication bias (bias coefficient = 0.137, *p* = 0.930), suggesting that heterogeneity can be explained by subgroup analysis and random-effects meta-analysis.

The sample was stratified based on post-ban duration (> 2 years versus ≤2 years), comprehensiveness of the smoke-free legislation (workplaces only versus workplaces, restaurants and bars), number of AMI deaths (< 10,000 versus ≥10,000), study location (Europe versus North America versus other locations), research classification (regional versus national) and previous ban in place (with prior local law versus without previous ban). The effects of smoke-free legislation on the AMI mortality rate under different circumstances were also calculated in this study.

**Fig. 4:**
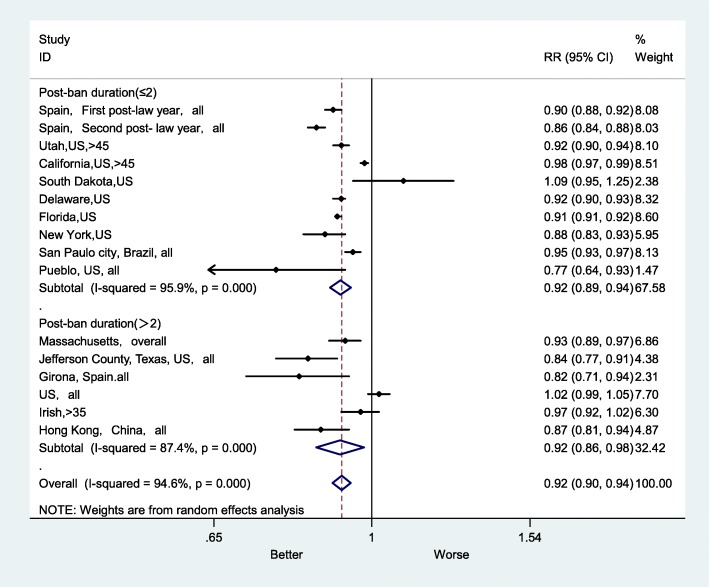
Forest plot of random effects meta-analysis of studies examining the effect of smoke-free legislation on AMI mortality stratified by post-ban duration

The implementation of smoke-free legislation could effectively reduce the mortality rate of AMI in the short and long term (Fig. 4). In the post-ban follow-up duration (≤2 years) subgroup, the RR was 0.92 (95% CI: 0.89–0.94). In the post-ban follow-up duration (> 2 years) subgroup, the RR was 0.92 (95% CI: 0.86–0.98), and I^2^ was 87.4% (*P* < 0.001). These results showed that the effect of smoke-free legislation on the AMI mortality rate was evident in both long-term and short-term studies.

**Fig. 5:**
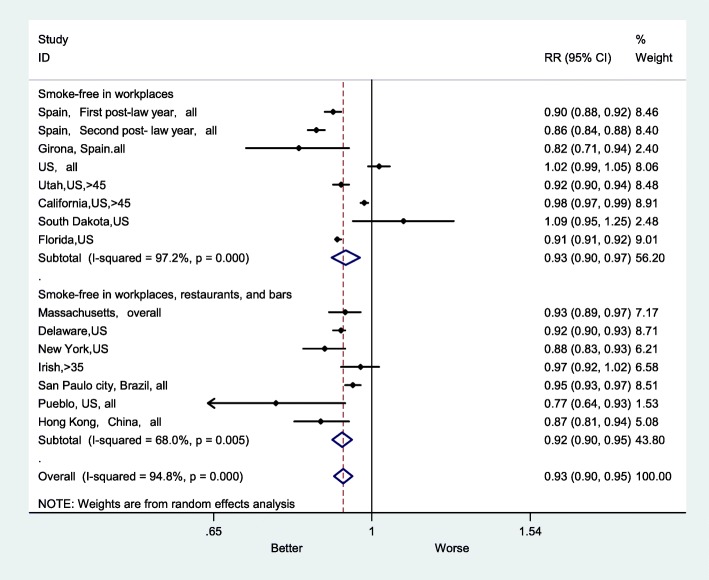
Forest plot of random effects meta-analysis of studies examining the effect of smoke-free legislation on AMI mortality stratified by comprehensiveness of a law

For 2 subgroups of different comprehensiveness levels of smoke-free legislation, the more comprehensive smoke-free legislation was significantly associated with a lower AMI mortality rate (Fig. 5). In the subgroup prohibiting smoking in workplaces, restaurants and bars, the RR was 0.92 (95% CI: 0.90–0.95), and I^2^ was 68.0% (*P* < 0.001). In the subgroup of prohibiting smoking in workplaces only, the RR was 0.93 (95% CI: 0.90–0.97) and I^2^ was 97.2% (P < 0.001).

**Fig. 6:**
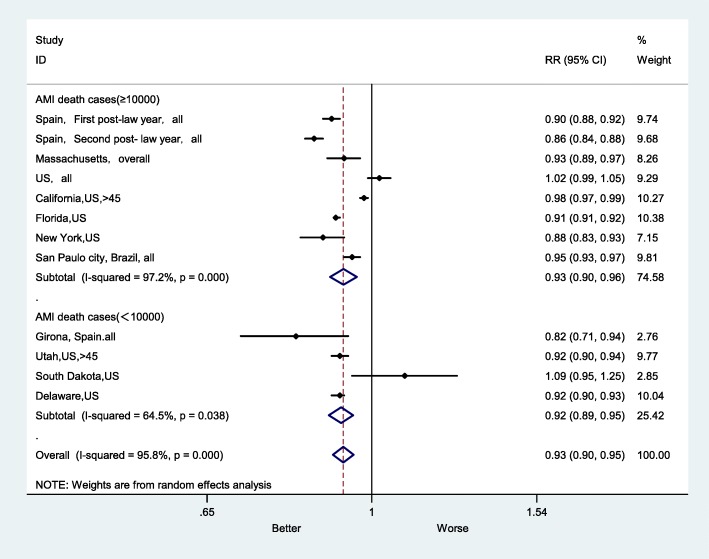
Forest plot of random effects meta-analysis of studies examining the effect of smoke-free legislation on AMI mortality stratified by AMI death cases

The RR for AMI mortality in the subgroups with larger numbers of AMI deaths was 0.93 (95% CI: 0.90–0.96), and the RR in the subgroups with smaller number of AMI death cases rate was 0.92 (95%CI: 0.89–0.95). I^2^ values were different in these 2 subgroups, 97.2 and 64.5%, respectively (Fig. 6).

**Fig. 7:**
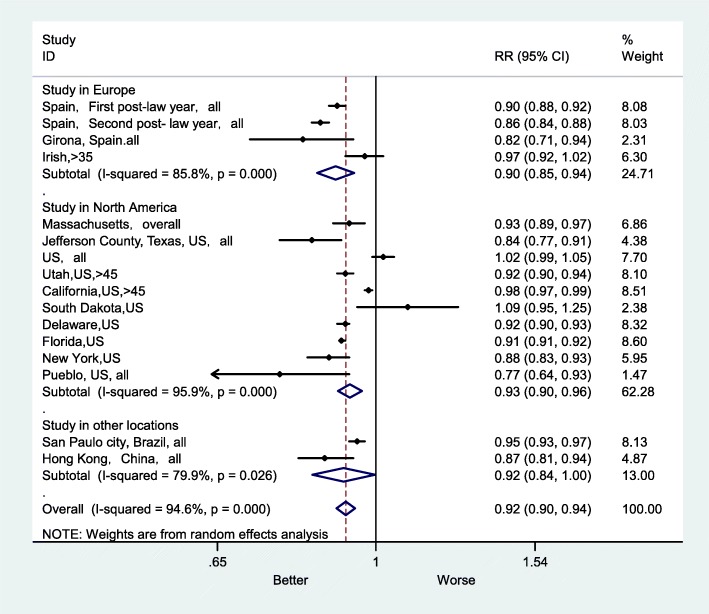
Forest plot of random effects meta-analysis of studies examining the effect of smoke-free legislation on AMI mortality stratified by study location

**Fig. 8:**
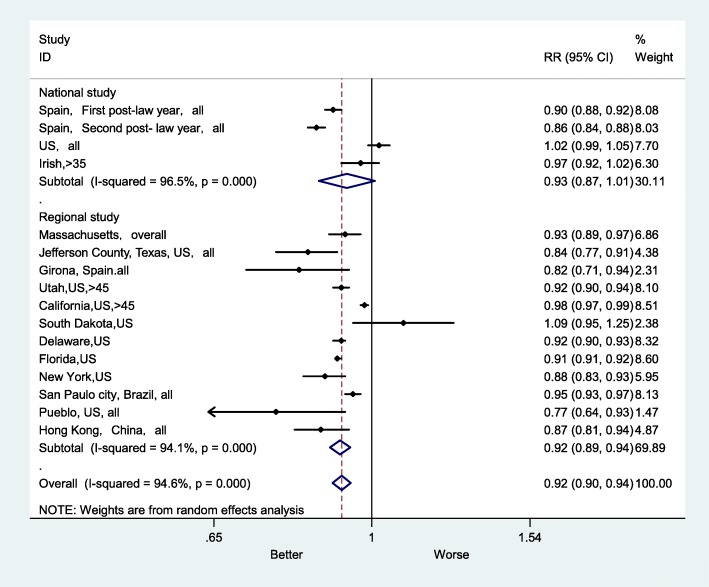
Forest plot of random effects meta-analysis of studies examining the effect of smoke-free legislation on AMI mortality stratified by study classification

The subgroup RRs for AMI mortality were 0.90 (95% CI: 0.85–0.94) in the European studies, 0.93 (95% CI: 0.90–0.96) in the US studies and 0.92 (95% CI: 0.84–1.00) in studies from other locations (Fig. 7). In other locations, the heterogeneity was lower (I^2^ = 79.9%, *P* < 0.026); however, the heterogeneity was higher in the US studies (I^2^ = 95.9%, *P* < 0.001). In addition, the subgroup RRs for AMI mortality were 0.93 (95% CI: 0.87–1.01) in national studies and 0.92 (95% CI: 0.89–0.94) in regional studies (Fig. 8). I^2^ was higher in national studies (I^2^ = 96.5%, P < 0.001), whereas it was lower in regional studies (I^2^ = 94.2%, P < 0.001).

**Fig. 9:**
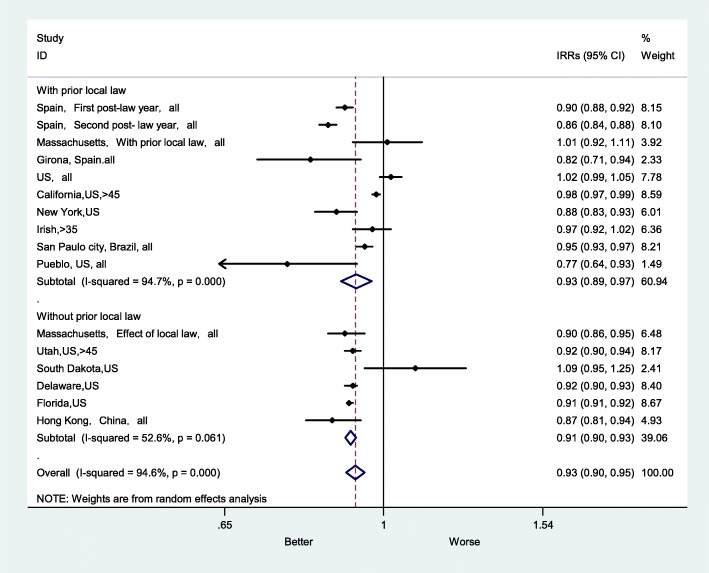
Forest plot of random effects meta-analysis of studies examining the effect of smoke-free legislation on AMI mortality stratified by previous ban in place. Note: studies without prior smoke-related bans were not included

The subgroup RRs for AMI mortality were higher in areas without prior local bans (RR = 0.91) (Fig. 9). In studies of areas with previous local bans, the impact of the smoke-free ban on AMI mortality RR were 0.93 (95% CI: 0.89–0.97), and the heterogeneity was relatively high (I^2^ = 94.7%, P < 0.001). In studies of areas without previous local bans, the heterogeneity was relatively low (I^2^ = 52.6%, *P* < 0.061).

**Fig. 10:**
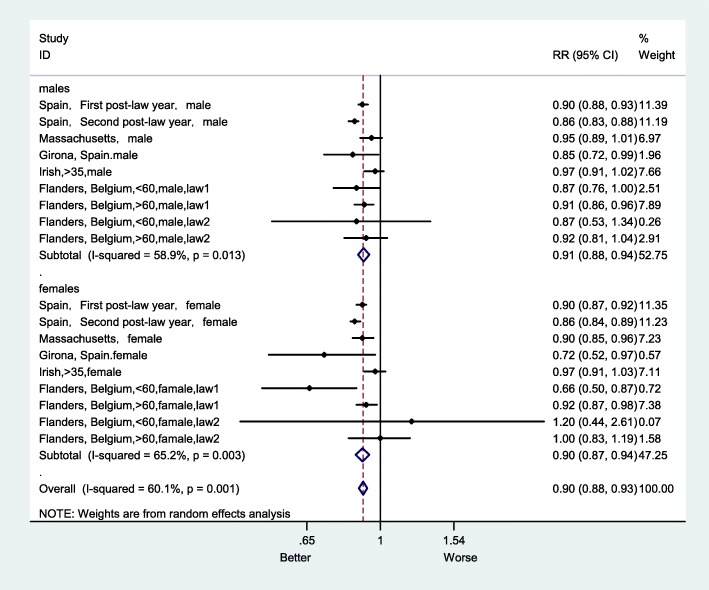
Forest plot of sensitivity analysis of studies examining the effect of smoke-free legislation on AMI mortality stratified by gender

**Fig. 11:**
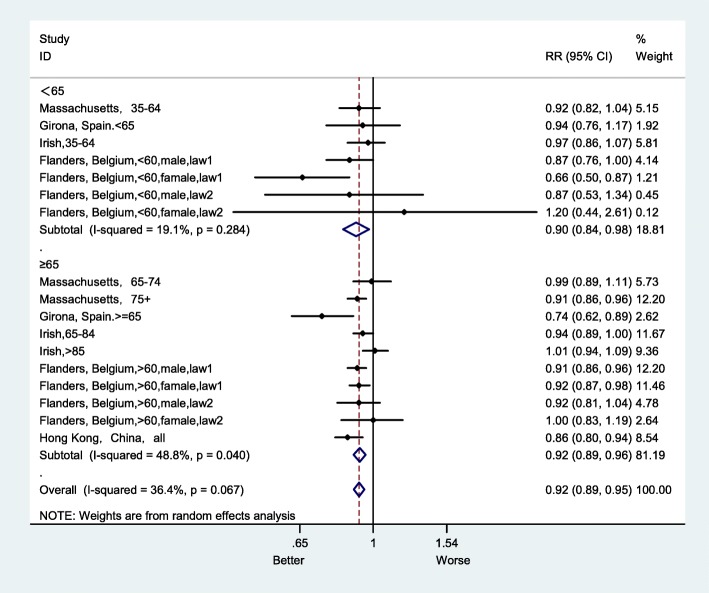
Forest plot of sensitivity analysis of studies examining the effect of smoke-free legislation on AMI mortality stratified by age

**Fig. 12:**
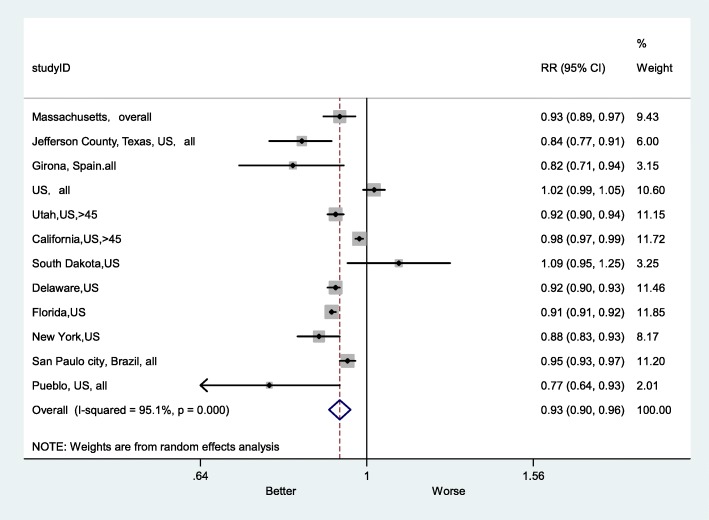
Forest plot of sensitivity analysis of studies examining the effect of smoke-free legislation on AMI mortality stratified after excluding studies with high risk bias

Sensitivity analysis, by sex, showed that in the female group, the impact of the smoke-free ban on AMI mortality RR was 0.90 (95% CI: 0.87–0.94), which was 1% higher than in male group (Fig. 10). In addition, the effect size of the RR for smoke-free legislation was 0.90 (95% CI:0.84–0.98) in the group aged less than 65, which was larger than the effect size for the group aged more greater than 65 (Fig. 11). After excluding studies with a high risk of bias, the overall RR was 0.93 (95% CI:0.90–0.96) (Fig. 12).

## Discussion

This systematic review and meta-analysis presented strong evidence concerning the effectiveness of smoke-free legislation on reducing the AMI mortality rate. Previous meta-analyses have estimated reductions in the incidence rate or hospital admission rate of the AMI after the implementation of smoke-free legislations [28–30]. To the best of our knowledge, this may be the first study to examine the relationship between AMI mortality rate and the corresponding smoke-free legislation.

The results of this study demonstrated that smoke-free legislation were associated with an 8% decrease in the AMI mortality rate in general. Furthermore, the degree of the decline in risk of AMI varied across different subgroups (2–23%). The AMI mortality rate decreased greatly among females (RR, 0.90; 95% CI: 0.87–0.94) and people aged ≤65 (RR, 0.90; 95% CI: 0.84–0.98). The different beneficial effects of legislation may be attributed to the diversity of study designs, including differences in the target populations, statistical analyses, types of smoke-free legislations, and differences in compliance with the law. [31]. There were 2 studies that did not find that smoke-free legislation was associated with a lower AMI mortality rate [15, 32]. Different methodology concerning the coverage of local smoke-free legislations and the time during which the bans were in effect may result in contradicting results for AMI mortality.

The largest effect of smoke-free legislation on the AMI mortality rate was found in.

places with more comprehensive bans [14, 16, 21–23, 25]. Allowing smoking in designated smoking areas or in ventilated smoking rooms would not effectively prevent secondhand smoke exposure [33]. More comprehensive smoke-free legislation could significantly reduce the number of active smokers [34], raise people’s awareness about the side effects of smoking, and more importantly, change social norms about the perception of smoking. Moritsugu noted that enacting more comprehensive tobacco-control legislation could effectively prevent exposure to secondhand smoke and reduce the number of smokers [35]. Based on European studies, Ward found that the indoor PM2.5 concentration generally decreased by 68.4%, while for areas with partial bans on smoking, indoor PM2.5 concentration reduced by 40%, after introducing comprehensive smoke-free legislation [36]. This provided sufficient evidence that enacting comprehensive smoke-free legislation was associated with lower level of exposure to secondhand smoke.

In this study, larger estimates of the effect were found in the smaller samples rather than in the larger samples, which was consistent with the results of a previous meta-analysis [37]. In studies of small samples, the RR values may be more sensitive to random factors; in our study, the standard deviation of the mean RR values was 0.11, which was higher than that of the larger samples (SD = 0.05).

Although some studies (with a post-ban duration ≤2 years) indicated that smoke-free legislation could result immediate effects on the AMI mortality rate [14, 17, 22, 25], the present study showed that the impacts of smoke-free legislation could increase over time. Four studies suggested that the effect of smoke-free legislation on the AMI mortality rate increased with a longer follow up period [14, 17, 20, 24]. Dove believes that the smaller reduction in AMI deaths after the legislation maybe because we examined AMI mortality rates were what we examined rather than hospitalization rates; therefore, it may take a longer time to reveal an immediate downward trend [14]. Figure 4 shows that studies with a longer follow-up period have smaller heterogeneity in the risk estimates than those in studies with a shorter follow up period.

The present study also showed that the decline in the AMI mortality rate was also associated with the situation where there were no smoking control measures before the smoke-free legislation [14, 16, 23]. The effect of smoke-free legislation may have been weakened when people had been protected by pre-existing local regulations. For example, California had developed local smoke-free measures before introducing nationwide smoke-free legislation, and almost 70% of the state’s population was protected [38]. Enacting smoking control measures could reduce secondhand exposure to some extent, which may minimize the effect size of implementing smoke-free legislation on the AMI mortality rate [23].

Subgroup analyses were performed to explore the effect of smoke-free legislation on AMI mortality by sex and age (as shown in Figs. 10 and 11). The decline in the AMI mortality rate in the female group was 10%, which was 1% higher than that in the male group (Fig. 10). In addition, there was also a decrease of 10% in the group with aged less than 65, which was larger than that in the group with aged greater than 65 (Fig. 11). Furthermore, a sensitivity analysis was performed, after excluding studies with a high risk of bias (as shown in Fig. 12), to ensure the robustness of the analysis, and the overall RR of AMI mortality was 0.93 (95% CI: 0.90–0.96), there was no appreciable change.

It is worth noting that 2 studies, lacking estimates for the whole target population, could not be directly used for meta-analysis of the whole target population. However, in our sensitivity analyses, we included in the analysis from a study in Flanders stratified by sex and age, and from another study in Rome stratified by age [32, 39]. The results of the sensitivity analysis showed that smoke-free legislation resulted in larger effects on AMI mortality rate within the female group [14, 20] and the younger people group (aged less than 65) [14, 17, 32]. One plausible explanation is that prohibiting smoking in the workplace could reduce the smoking prevalence in work areas among young working populations [37]. In addition, the older people are less likely to go to bars and similar venues, which could lead to smaller relatives risks associated with secondhand smoke exposure in this age group people [23]. 35% of females and 33% of males were exposed to secondhand smoke [40], but the smoking prevalence among females was relatively lower compared to that among males. Therefore, after implementing smoke-free legislation, more females would be protected from secondhand smoke exposure, which would lead to fewer women dying of AMI.

Other smoking control measures after smoke-free legislation also influence the AMI mortality rate. Thach found that in Hong Kong, the increase in the tobacco tax strengthened the effectiveness of smoke-free legislation [23]. McAlister also estimated that the influence of raising the tobacco tax would be reflected in the smoking prevalence [24].

In this study, several limitations should be noted. First, we analyzed the change in the AMI mortality rate before and after implementing smoke-free legislation, but the causal relationship between them could not be explored. It was not possible to find a location that was identical and that did not have smoke-free legislation to include as a control group when we assessed the effects of smoke-free legislation on the AMI mortality rate. The data included in this study were all extracted from time series data to evaluate the potential effect of smoke-free legislation.

Second, during subgroup analysis, the comprehensiveness of the smoke-free legislation was entered into the model as an ordinal variable (0 for workplaces only; 1 for workplaces, restaurants and bars) to test whether comprehensive laws were more beneficial to the considerable decline in the AMI mortality rate. Hence, the expected decrease in risk per 1% or per 1-standard deviation (SD) decrease in the AMI mortality rate, in the meta-analysis, could not be calculated.

Third, studies included in this meta-analysis did not account for the nonlinear trend in the AMI mortality rate, which might be concern in our estimates. The nonlinear secular trend could be explained by the concomitant effect of other time-varying factors [41], only 2 studies compared models with different specifications of secular trend and showed that the estimated effect was attenuated under the condition of nonlinearity in the secular trend of declining AMI [22, 41] .

Fourth, inaccuracies might have existed due to the lack of control for other confounding factors. Some studies included in this meta-analysis only considered individual factors, such as sex and age, and few studies have considered air quality. However, other studies have documented that environmental factors such as air temperature, air pressure, and air quality were related to AMI mortality rates [42–44]. Furthermore, technological advances in medicine [45] and increases in tobacco taxes and prices would also influence the AMI mortality rate [46]. In this meta-analysis, 2 studies did not consider confounding factors [16, 24].

Despite the methodological limitations of the individual studies included, the present study still provided evidence-based assessments of the effect of smoke-free legislation on AMI mortality rates around the world. These results may provide evidence for promoting smoke-free legislation in areas that allow indoor and outdoor smoking.

## Conclusion

This study provided evidence that a larger decline in the AMI mortality rate was found after smoke-free legislation. Comprehensive laws ending smoking in workplaces, restaurants, and bars, assessment at the regional level, a study location in Europe, the lack of established policies prior to the legislation, and smaller sample sizes were associated with greater effects. However, there was no difference in the effect of smoke-free legislation on AMI mortality rates in between the longer follow-up duration and shorter. Countries should be strongly encouraged to introduce more comprehensive smoke-free legislation applies to both public areas and workplaces.

## Additional file

Additional file 1: Search Phrases for a) PubMed, and b) EMBASE, and c) Web of Science, and d) Google Scholar. (DOC 49 kb)

## Data Availability

The datasets analyzed during this study are available from the corresponding author on reasonable request.

## Abbreviations

AMI: Acute myocardial infarction
DALYs: Disability-adjusted life year
GBD: Global Burden of Disease Study
IARC: International Agency for Research on Cancer
RR: Relative risk

## References

[1] Organization WH: WHO report on the global tobacco epidemic, 2013. Enforcing bans on tobacco advertising, promotion and sponsorship 2013.

[2] Reitsma MB, Fullman N, Ng M, Salama JS, Abajobir A, Abate KH, Abbafati C, Abera SF, Abraham B, Abyu GY (2017). Smoking prevalence and attributable disease burden in 195 countries and territories, 1990–2015: a systematic analysis from the Global Burden of Disease Study 2015. Lancet.

[3] Barnoya J, Glantz SA (2005). Cardiovascular effects of secondhand smoke: nearly as large as smoking. Circulation.

[4] Öberg M, Jaakkola MS, Woodward A, Peruga A, Prüss-Ustün A (2011). Worldwide burden of disease from exposure to second-hand smoke: a retrospective analysis of data from 192 countries. Lancet.

[5] U.S. Department of Health and Human Services : How tobacco smoke causes disease: The biology and behavioral basis for smoking-attributable disease: A report of the surgeon general. Atlanta, GA: U.S. Department of Health and Human Services, Centers for Disease Control and Prevention, National Center for Chronic Disease Prevention and Health Promotion, Office on Smoking and Health, 2010. 21452462

[6] Dunbar A, Gotsis W, Frishman W (2013). Second-hand tobacco smoke and cardiovascular disease risk: an epidemiological review. Cardiol Rev.

[7] Forouzanfar MH, Alexander LT, Anderson HR, Bachman VF, Biryukov S, Brauer M, Burnett RT, Casey DC, Coates MM, Cohen A (2015). Global, regional, and national comparative risk assessment of 79 behavioural, environmental and occupational, and metabolic risks or clusters of risks in 188 countries, 1990-2013: a systematic analysis for the global burden of disease study 2013. Lancet.

[8] Elkhader BA, Abdulla AA, Ali Omer MA (2016). Correlation of smoking and myocardial infarction among Sudanese male patients above 40 years of age. Pol J Radiol.

[9] Oliveira A, Barros H, Maciel MJ, Lopes C (2007). Tobacco smoking and acute myocardial infarction in young adults: a population-based case-control study. Prev Med.

[10] Negri E, La Vecchia C, Nobili A, D'Avanzo B, Bechi S (1994). Cigarette smoking and acute myocardial infarction. A case-control study from the GISSI-2 trial. GISSI-EFRIM Investigators. Gruppo Italiano per lo Studio della Sopravvivenza nell'Infarto--Epidemiologia dei Fattori di Rischio dell'infarto Miocardioco. Eur J Epidemiol.

[11] Nyboe J, Jensen G, Appleyard M, Schnohr P (1991). Smoking and the risk of first acute myocardial infarction. Am Heart J.

[12] Hu TW, Lee AH, Mao Z (2013). WHO framework convention on tobacco control in China: barriers, challenges and recommendations. Glob Health Promot.

[13] Tan CE, Glantz SA (2012). Association between smoke-free legislation and hospitalizations for cardiac, cerebrovascular, and respiratory diseases: a meta-analysis. Circulation.

[14] Dove MS, Dockery DW, Mittleman MA, Schwartz J, Sullivan EM, Keithly L, Land T (2010). The impact of Massachusetts’ smoke-free workplace Laws on acute myocardial infarction deaths. Am J Public Health.

[15] Shetty KD, Deleire T, White C, Bhattacharya J (2011). Changes in U.S. hospitalization and mortality rates following smoke-free legislations. J Policy Anal Manage.

[16] Rodu B, Peiper N, Cole P (2011). Acute myocardial infarction mortality before and after state-wide smoke-free legislations. J Community Health.

[17] Villalbí JR, Sánchez E, Benet J, Cabezas C, Castillo A, Guarga A, Saltó E, Tresserras R (2011). The extension of smoke-free areas and acute myocardial infarction mortality: before and after study. BMJ Open.

[18] Cancer IAfRo. Evaluating the effectiveness of smoke-free policies: Organization WH; 2009.

[19] Tan CE, Glantz SA (2012). Association between Smokefree legislation and hospitalizations for cardiac, cerebrovascular and respiratory diseases: a Meta-analysis. Circulation.

[20] Agüero F, Dégano IR, Subirana I, Grau M, Zamora A, Sala J, Ramos R, Treserras R, Marrugat J, Elosua R (2013). Impact of a partial smoke-free legislation on myocardial infarction incidence, mortality and case-fatality in a population-based registry: the REGICOR study. PLoS One.

[21] Stallingssmith S, Zeka A, Goodman P, Kabir Z, Clancy L (2013). Reductions in cardiovascular, cerebrovascular, and respiratory mortality following the National Irish Smoke-free legislation: interrupted time-series analysis. PLoS One.

[22] Bartecchi C, Alsever RN, Nevin-Woods C, Thomas WM, Estacio RO, Bartelson BB, Krantz MJ (2006). Reduction in the incidence of acute myocardial infarction associated with a citywide smoking ordinance. Circulation.

[23] Thach T-Q, McGhee SM, So JC, Chau J, Chan EK, Wong C-M, Hedley AJ (2015). The smoke-free legislation in Hong Kong: its impact on mortality. Tob Control.

[24] Mcalister AL, Huang P, Ramirez AG, Harrist RB, Fonseca VP (2010). Reductions in cigarette smoking and acute myocardial infarction mortality in Jefferson County, Texas. Am J Public Health.

[25] Abe TM, Scholz J, De ME, Nobre MR, Filho RK. Decrease in mortality rate and hospital admissions for acute myocardial infarction after the enactment of the smoke-free legislation in São Paulo city, Brazil. Tob Control. 2017;26:656–62. 10.1136/tobaccocontrol-2016-053261 27794066

[26] Effective Practice and Organization of Care (EPOC): EPOC Resources for review authors. http://epoc.cochrane.org/resources/epoc-resources-review-authors. Accessed 26 Oct 2017.

[27] Sterne JAC, Sutton AJ, Ioannidis JPA, Terrin N, Jones DR, Lau J, Carpenter J, Rücker G, Harbord RM, Schmid CH (2011). Recommendations for examining and interpreting funnel plot asymmetry in meta-analyses of randomised controlled trials. BMJ.

[28] Glantz SA (2008). Meta-analysis of the effects of smokefree laws on acute myocardial infarction: an update. Prev Med.

[29] Meyers DG, Neuberger JS, He J (2009). Cardiovascular effect of bans on smoking in public places: a systematic review and meta-analysis. J Am Coll Cardiol.

[30] Lin H, Wang H, Wu W, Lang L, Wang Q, Tian L (2013). The effects of smoke-free legislation on acute myocardial infarction: a systematic review and meta-analysis. BMC Public Health.

[31] Barr CD, Diez DM, Wang Y, Dominici F, Samet JM (2012). Comprehensive smoke-free legislations and acute myocardial infarction among Medicare enrollees in 387 US counties: 1999–2008. Am J Epidemiol.

[32] Cox B, Vangronsveld J, Nawrot TS (2014). Impact of stepwise introduction of smoke-free legislation on population rates of acute myocardial infarction deaths in Flanders, Belgium. Heart.

[33] Health UDo, Services H: the health consequences of involuntary exposure to tobacco smoke: a report of the surgeon general. Atlanta, GA: US Department of Health and Human Services, Centers for Disease Control and Prevention, Coordinating Center for Health Promotion, National Center for Chronic Disease Prevention and Health Promotion, Office on Smoking and Health 2006, 709. 20669524

[34] Arnott D, Dockrell M, Sandford A, Willmore I (2007). Comprehensive smoke-free legislation in England: how advocacy won the day. Tob Control.

[35] Moritsugu KP (2007). The 2006 report of the surgeon general : the health consequences of involuntary exposure to tobacco smoke. Am J Prev Med.

[36] Ward M, Currie LM, Kabir Z, Clancy L (2013). The efficacy of different models of smoke-free laws in reducing exposure to second-hand smoke: a multi-country comparison. Health Policy.

[37] Fichtenberg CM, Glantz SA (2002). Effect of smoke-free workplaces on smoking behaviour: systematic review. Bmj.

[38] Health UDo (1993). Health HSNIo: Major local tobacco control ordinances in the United States. Smoking & Tobacco Control Monographs.

[39] Cesaroni G, Forastiere F, Agabiti N, Valente P, Zuccaro P, Perucci CA (2008). Effect of the Italian smoke-free legislation on population rates of acute coronary events. Circulation.

[40] Musk AW, De Klerk NH (2003). History of tobacco and health. Respirology.

[41] Gasparrini A, Gorini G, Barchielli A (2009). On the relationship between smoke-free legislations and incidence of acute myocardial infarction. Eur J Epidemiol.

[42] Hopstock LA, Wilsgaard T, Njølstad I, Mannsverk J, Mathiesen EB, Løchen ML, Bønaa KH: Seasonal variation in incidence of acute myocardial infarction in a sub-Arctic population: the Tromsø study 1974-2004. European journal of cardiovascular prevention and rehabilitation: official journal of the European Society of Cardiology, Working Groups on Epidemiology & Prevention and Cardiac Rehabilitation and Exercise Physiology 2011, 18(2):320–325. 10.1097/HJR.0b013e32833c7c28 20606596

[43] Kriszbacher I, Boncz I, Koppán M, Bódis J (2008). Seasonal variations in the occurrence of acute myocardial infarction in Hungary between 2000 and 2004. Int J Cardiol.

[44] Lee JH, Chae SC, Dong HY, Park HS, Cho Y, Jun JE, Park WH, Kam S, Lee WK, Kim YJ (2009). Influence of weather on daily hospital admissions for acute myocardial infarction (from the Korea acute myocardial infarction registry). Int J Cardiol.

[45] Weisfeldt ML, Zieman SJ (2007). Advances in the prevention and treatment of cardiovascular disease. Health Aff.

[46] Ho V, Ross JS, Steiner CA, Mandawat A, Short M, Ku-Goto MH, Krumholz HM (2016). A Nationwide assessment of the Association of Smoke-free legislations and cigarette taxes with hospitalizations for acute myocardial infarction, heart failure, and pneumonia. Med Care Res Rev.

