# Supplementary material for: The effect of smoke-free legislation on the mortality rate of acute myocardial infarction: a meta-analysis
Source: BMC Public Health. 2019 Sep 18;19:1269. doi: 10.1186/s12889-019-7408-7 (PMC6749716; doi:10.1186/s12889-019-7408-7)
Supplement: Supplementary file 1 — Search Phrases for a) PubMed, and b) EMBASE, and c) Web of Science, and d) Google Scholar. (DOC 49 kb) [file 12889_2019_7408_MOESM1_ESM.doc]

**Additional file 1. Search Phrases for a) PubMed, and b) EMBASE, and c) Web of Science, and d) Google Scholar**

a) Search strategy for PubMed (Publication date to 2017/9/30)

| 1. " smoke-free laws"[Title/Abstract] |
| --- |
| 1. ((Smoke law[Title/Abstract]) OR Smoke legislation[Title/Abstract]) OR Smoke ban[Title/Abstract] OR smoke-free laws[Title/Abstract]) OR tobacco control law[Title/Abstract] |
| 1. 1 OR 2 |
| 1. ((((Acute myocardial infarction *[Title/Abstract]) OR Cardiovascular *[Title/Abstract]) OR Coronary *[Title/Abstract]) |
| 1. ((Mortality *[Title/Abstract]) OR Death *[Title/Abstract]) |
| 1. 4 AND 5 |
| 1. 3 AND 6 |

**((((((((Acute myocardial infarction *[Title/Abstract]) OR Cardiovascular *[Title/Abstract]) OR Coronary *[Title/Abstract]))) OR (((Mortality *[Title/Abstract]) OR Death *[Title/Abstract])))) AND ((" smoke-free laws") OR (((Smoke law[Title/Abstract]) OR Smoke legislation[Title/Abstract]) OR Smoke ban[Title/Abstract] OR smoke-free laws[Title/Abstract]) OR tobacco control law[Title/Abstract]))**

**N=375**

b) Search strategy for EMBASE (Publication date to 2017/9/30)

| 1. "smoke-free" or "smoke law" or "Smoke legislation" or " tobacco control " |
| --- |
| 1. (Acute myocardial infarction ' OR ' Cardiovascular ' OR Coronary ' |
| 1. Mortality OR' Death |
| 1. 1 AND 2 AND 3 |

TITLE-ABSTR-KEY(smoke-free) and TITLE-ABSTR-KEY(acute myocardial infarction Mortality ).N=6

TITLE-ABSTR-KEY ("smoke law " ) and TITLE-ABSTR-KEY ("acute myocardial infarction") and TITLE-ABSTR-KEY (" Mortality").N=0

TITLE-ABSTR-KEY(smoke ban ) and TITLE-ABSTR-KEY(acute myocardial infarction Mortality )N=2

TITLE-ABSTR-KEY(smoke legislation) and TITLE-ABSTR-KEY(acute myocardial infarction Mortality ).N=1

TITLE-ABSTR-KEY(tobacco control) and TITLE-ABSTR-KEY(acute myocardial infarction Mortality ).N=6

TITLE-ABSTR-KEY(smoke-free) and TITLE-ABSTR-KEY(acute myocardial infarction Death).N=12

TITLE-ABSTR-KEY(smoke law) and TITLE-ABSTR-KEY(acute myocardial infarction Death). N=0

TITLE-ABSTR-KEY(smoke ban) and TITLE-ABSTR-KEY(acute myocardial infarction Death). N=0

TITLE-ABSTR-KEY(smoke legislation) and TITLE-ABSTR-KEY(acute myocardial infarction Death). N=0

TITLE-ABSTR-KEY(tobacco control) and TITLE-ABSTR-KEY(acute myocardial infarction Death). N=5

TITLE-ABSTR-KEY("smoke-free" or "smoke law" or "Smoke legislation" or " tobacco control ") and TITLE-ABSTR-KEY(acute myocardial infarction Death). N=2

TITLE-ABSTR-KEY ("smoke-free" or "smoke law" or "Smoke legislation" or " tobacco control ") and TITLE-ABSTR-KEY(Cardiovascular Mortality ). N=16

TITLE-ABSTR-KEY("smoke-free" or "smoke law" or "Smoke legislation" or " tobacco control ") and TITLE-ABSTR-KEY(Coronary Mortality ). N=7

TITLE-ABSTR-KEY ("smoke-free" or "smoke law" or "Smoke legislation" or " tobacco control ") and TITLE-ABSTR-KEY(Cardiovascular death ). N=9

TITLE-ABSTR-KEY("smoke-free" or "smoke law" or "Smoke legislation" or " tobacco control ") and TITLE-ABSTR-KEY(Coronary death). N=2

N=68

c) Search strategy for **Web of Science** (Publication date to 2017/9/30)

| 1. "smoke-free" or "smoke law" or "Smoke legislation" or " tobacco control " |
| --- |
| 1. Acute myocardial infarction |
| 1. "Mortality" OR "Death" |
| 1. 1 AND 2 AND 3 |

("smoke-free" or "smoke law" or "Smoke legislation") AND (acute myocardial infarction) AND ( " Mortality" or "death")

N=36

d) Search strategy for **Google Scholar** (Publication date to 2017/9/30)

| 1. "smoke-free or smoke law or Smoke legislation or tobacco control " |
| --- |
| 1. "Acute myocardial infarction" |
| 1. "Mortality OR Death " |
| 1. 1 AND 2 AND 3 |

""smoke-free ban " OR "smoke legislation" OR "tobacco control"" AND "Acute myocardial infarction" AND ""Mortality" OR "Death ""

N=996

e) Search strategy for **CNKI** (Publication date to 2017/9/30)

| 1. "无烟立法" OR "控烟" |
| --- |
| 1. "急性心肌梗死" |
| 1. "死亡" OR "死亡率" OR "死" |
| 1. 1 AND 2 AND 3 |

("无烟立法" OR "控烟") AND ("急性心肌梗死" ) AND ("死亡" OR "死亡率" OR "死"

N=85
